# A global comprehensive review of economic interventions to prevent intimate partner violence and HIV risk behaviours

**DOI:** 10.1080/16549716.2017.1290427

**Published:** 2017-05-03

**Authors:** Andrew Gibbs, Jessica Jacobson, Alice Kerr Wilson

**Affiliations:** ^a^ Gender and Health Research Unit, South African Medical Research Council, Pretoria, South Africa; ^b^ Health Economics and HIV/AIDS Research Division (HEARD), University of KwaZulu-Natal, Durban, South Africa; ^c^ Social Development Direct, London, UK

**Keywords:** Globalisation, health information, health determinants, health intervention, population health

## Abstract

**Background**: Intimate partner violence (IPV) and HIV are co-occurring global epidemics, with similar root causes of gender and economic inequalities. Economic interventions have become a central approach to preventing IPV and HIV.**Objective/Methods**: We undertook a comprehensive scoping review of published evaluations of economic interventions that sought to prevent IPV and/or HIV risk behaviours.**Results**: Forty-five separate analyses of interventions met our criteria. Broadly, unconditional cash transfer interventions showed either flat or positive outcomes; economic strengthening interventions had mixed outcomes, with some negative, flat and positive results reported; interventions combining economic strengthening and gender transformative interventions tended to have positive outcomes.**Conclusions**: The review highlighted a number of gaps. Specifically, there were limited studies evaluating the impact of economic interventions on female sex workers, young women, and men. In addition, there were missed opportunities, with many evaluations only reporting either IPV- or HIV-related outcomes, rather than both, despite overlaps.

## Background

Intimate partner violence (IPV) and HIV acquisition are co-occurring global epidemics [1]. Globally the World Health Organization (WHO) [2] estimates that 30% of all women have experienced some form of sexual and/or physical violence from an intimate partner in their life. There is clear evidence that IPV is a major driver of HIV acquisition amongst heterosexual women [1,3] with studies suggesting up to 25% of all HIV acquisitions occurring amongst women are linked to their experiences of IPV [3,4]. In addition, HIV acquisition is also a cause of IPV [1]. The recognition of these linkages has led to a concerted effort by global institutions and researchers to design interventions to prevent IPV and HIV simultaneously [1,5–7], with programmes such as the Determined, Resilient, Empowered, AIDS-free, Mentored, and Safe women’s (DREAMS) initiative, a President’s Emergency Plan for AIDS Relief (PEPFAR)-led programme in six countries across southern and eastern Africa, channelling significant money into tackling HIV acquisition through reducing women’s experiences of IPV, with a particular focus on adolescents.

**Figure 1.**
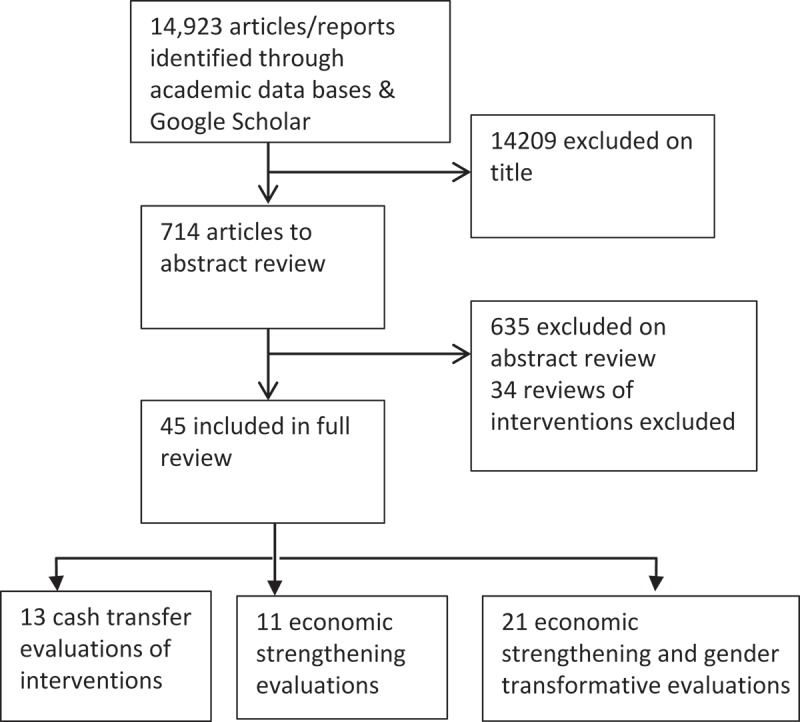
Flow chart of search.

Studies highlight the overlapping drivers of IPV- and HIV-vulnerability, particularly poverty and gender inequalities [6,8–10]. There is overwhelming evidence that gender inequalities shape men’s perpetration of IPV and women’s experience of IPV as well as their vulnerability to HIV in heterosexual relationships [3,11–14]. The evidence linking poverty and HIV-vulnerability is less clear, with recent longitudinal studies suggesting poverty can either be a risk or protective factor for HIV depending on other social factors including community acceptability of violence against women [15]. There is stronger evidence about the impact of poverty on women’s experiences of IPV. While it is certainly clear that IPV is a global phenomenon experienced by women in high-income as well as low-income countries [2], studies consistently show that women’s recent experience of IPV is strongly associated with their experiences of poverty [16–18].

Less contentious is the argument that poverty, when intersecting with gender inequalities, places women in economically and socially dependent relationships with men, which increases their vulnerability to HIV and IPV. This exacerbates the challenges of negotiating condoms, leaving violent and controlling relationships and exposing them to controlling behaviours: all risk factors for HIV acquisition and experiencing IPV [8–10]. More recently for men, qualitative research has argued that men’s partial exclusion from the capitalist economy has led men to develop identities that draw on and deploy forms of emphasised heterosexuality (occassionaly labelled a ‘hyper-masculinity’), which include control and domination over women, including IPV as a way to control women, as well as seeking multiple sexual partners [19–21].

In recognising how poverty shapes IPV and HIV-vulnerability, there has been significant research around using economic interventions to prevent HIV and IPV [6,22–24]. Broadly there are three conceptual approaches. The first is a social protection framework and the direct transfer of resources to households in the form of either cash or food/vouchers [25]. Transfers can be unconditional, or conditional on recipients accessing services or similar, and target poverty as an ‘upstream’ driver of ill-health [26]. A second approach is located within behavioural economics and uses direct cash transfers to incentivise certain behaviours or outcomes, be this HIV-testing or remaining Sexually Transmitted Infections (STI)-free [26,27]. These assume people make decisions based on trade-offs between alternatives and that the economic incentives are large enough to change behaviours in positive directions [26]. A third approach focuses on supporting those targeted to develop their own economic assets through either extending savings and loans systems to poor populations (e.g. microfinance) or providing vocational training [22]. A subset of these include gender transformative programming at the same time as economic strengthening [6]. These combined interventions are often focused on challenging women’s social and economic dependency on men.

There has been recent interest in economic interventions to prevent HIV acquisition and IPV, particularly in the context of UNAIDS’ Investment Framework [28,29]. Currently there exist a number of reviews of this evidence base, but these have been limited in scope. Reviews have focused only on economic interventions [22], or only on HIV outcomes or IPV outcomes, or have been of limited geographical scope [6,24,25]. None have sought to review any economic interventions including combined economic and gender transformative interventions and include HIV and/or IPV as outcomes. In this paper we undertook a comprehensive review of economic interventions globally that seek to prevent HIV or IPV.

## Methods

We conducted a comprehensive review of published and grey literature. We searched the formal academic websites PubMed, Web of Science and EbscoHost and searched grey literature using Google Scholar. The search terms covered (1) IPV, (2) HIV, (3) economic interventions (the full search string is given in the Appendix). We also used a snowballing technique to search reference lists and review articles to identify other studies.

Articles were included in the review if: (1) they had a quantitative evaluation of an intervention, whatever the study design (including cross-sectional, quasi-experimental and randomised control trial [RCT]); (2) they reported an outcome of either IPV or HIV risk behaviour – HIV risk behaviours had to be behavioural such as condom use at last sex, transactional sex and number of partners; (3) the intervention included an economic component; (4) they were published in English between 1 January 2000 and 1 January 2015.

Exclusion criteria were: (1) qualitative evaluations; (2) they only reported knowledge and/or intentions around HIV (rather than a behavioural outcome); (3) they fell outside of the date range for publication; (4) they were reviews synthesising other studies. There were no exclusion criteria based on quality of study design. The lack of inclusion of qualitative research means that there cannot be a focus on the processes of change in the review.

Initial searches were conducted on relevant databases with all references downloaded to EndNote 7 for review. Articles were initially screened based on title by the first author. A more comprehensive review of abstracts was then conducted. Full texts were read if clarity was further needed (see Figure 1). Data was extracted into a spreadsheet to ensure consistency of reporting.

Where a number of separate analyses sought to understand the impact of the same intervention (for instance multiple analyses of the Child Support Grant [CSG] in South Africa), these are reported separately. We provide a narrative review of the evidence.

## Results

In total we identified 45 separate analyses of interventions meeting our criteria (see Figure 1). Given the heterogeneity in interventions we categorised interventions into three categories based on their economic intervention type: (1) evaluations of cash transfer interventions (n = 13; see Table 1); (2); evaluations of economic strengthening interventions (n = 11; see Table 2); and (3) and evaluations of economic strengthening and gender transformative interventions (n = 21; see Table 3). Overall, only eight interventions were reported in the grey literature.

1. Cash transfer interventions

**Table 1.** Cash transfer interventions to prevent HIV or IPV (1 January 2000–1 January 2015).

Reference	Intervention	Country	Population	Study design	HIV outcome	IPV outcome
Social protection cash transfers – child-focused outcomes						
Cluver et al. [30]	Child Support Grant (CSG) – paid to caregiver; conditional on low income; value ZAR 250/month in 2010 and ZAR 280/month in 2012; roughly equivalent to US.$ 35	South Africa	Adolescents 10–18	3515 participants; prospective study; 1-year follow-up; propensity score matching	For adolescent girls receipt of cash transfer associated with: * Reduced incidence of transactional sex (odds ratio [OR] 0.49, 95% CI 0.26–0.93; *p* = 0.028) * Reduced incidence of age-disparate sex (OR 0.29, 95% CI 0.13–0.67; *p* = 0.004) * No significant effects for boys	
Cluver et al. [31]	CSG; child-focused care (naturally occurring)	South Africa	Adolescents 10–18	3515 participants; prospective study; 1-year follow-up	* Combined cash plus care reduced HIV risk behaviour for girls (OR 0.55; 95% CI 0.35–0.85; *p* = 0.007) and boys (OR 0.50; 95% CI 0.31–0.82; *p* = 0.005)	
UNICEF [32]	CSG – paid to caregiver; conditional on low income; value ZAR 250/month in 2010 and ZAR 280/month in 2012; roughly equivalent to US.$ 35	South Africa	Children (10 years old) and adolescents (15–17)	716 children; 1726 adolescents; matched participants on early or late entry to CSG; cross-sectional	* Reduced sexual activity and fewer number of partners (signif) * Reduced pregnancy (signif) * Reduced alcohol and drug use (signif)	
Handa et al. [33]	Cash Transfer for Orphans and Vulnerable Children (Kenya CT-OVC): Unconditional transfer US.$ 20/month	Kenya	Young people 15–25	1540 intervention and 754 control households; 28 areas randomly allocated to intervention/control arms; 2-year follow-up	* Reduced sexual debut by 31% (adjusted odds ratio [AOR] 0.69; 95% CI 0.53–0.89) * No statistically significant effects on condom use, number of partners and transactional sex	
Rosenberg et al. [34]	Kenya CT-OVC: Unconditional transfer US.$ 20/month	Kenya	Young people 15–25	1540 intervention and 754 control households; 28 areas randomly allocated to intervention/control arms; 2-year follow-up	* No significant impact on relative partner age, partner school status or transactional sex for young women or men Point estimates on transactional sex were on opposite sides of the null for men and women though not significant: * Men in intervention reported higher rates of transactional sex than control (a.OR = 1.57; 95% CI 0.60–4.07; v2 = 0.85; *p* = 0.36) * Women reported less transactional sex than women in the control arm (a.OR = 0.79; 95% CI 0.40–1.58; v2 = 0.55; *p* = 0.51), with greater effects for younger women (a.OR: 0.65; 95% CI 0.30–1.42; v2 = 1.18; *p* = 0.28) and those currently enrolled in school (a.OR: 0.38; 95% CI 0.13–1.11; v2 = 3.15; *p* = 0.08)	
**Social protection cash transfers – adult-focused outcomes**						
Hidrobo [35]	Bono de Desarrollo Humano (BDH); means tested; unconditional; US.$ 15/month	Ecuador	Poor families identified through means test	118 parishes randomly assigned, 79 to intervention and 39 to control; 2354 interviews; 24-month follow-up		* Intervention had no effect on emotional and physical violence * Intervention had negative impact on controlling behaviours (*p* < 0.05)
Hidrobo [36]	6-monthly transfer of cash, vouchers or food of U.S.$ 40/month (total U.S.$ 240)	Ecuador	Women	2357 randomised into intervention and control arms; 6-month follow-up		*Intervention reduces controlling behaviors, moderate physical, and any physical/sexual violence by 6 to 7 percentage points (*p* < 0.05)
Bobonis [37]	Oportunidades: conditional grants; food grant; school scholarship; range of values, max 625 pesos/month	Mexico	Poor women	54,000 households; national household survey		* Intervention reduces physical violence by 40% (*p* < 0.01) * Intervention increases by 3–5% points emotional violence (not significant)
Bobonis [38]	Oportunidades: conditional grants; food grant; school scholarship; range of values, max 625 pesos/month	Mexico	Poor women	54,000 households; national household survey		* 5–9 years later no differences on physical and emotional violence * Potential that women left violent relationships
Haushofer [39]	Unconditional cash transfer; either to husband or wife; either lump sum or nine monthly sums; values varied (either US $300 or US $1100)	Kenya	General population	990 households; randomised 3 arms (and more internally); 18-month follow-up		* 30–50% reduction in various forms of physical violence * 50% reduction in rape (*p* < 0.1) and 60% reduction in other forms of sexual violence (*p* < 0.05)
**Conditional cash transfers for HIV prevention**						
de Walque [40]	Conditional cash transfer (low: US.$ 10/test, high: US.$ 20/test) every 4 months for 12 months	Tanzania	Young women and men	2399 randomised into control, low or high incentive; 12-month follow-up	* Adjusted analyses found high value incentive led to significant reduction in STIs (adjusted risk ratio [a.RR] was 0.73) * No significant changes in low-value incentives	
Kohler and Thornton [41]	Malawi Incentives Project: conditional cash transfer on maintaining HIV-status for one year; reward ranged from zero to approximately 4 months’ wages	Malawi	Male and female adults	1307 randomised into value of incentives; 12-month follow-up	* Incentives had no impact on HIV-status or reported sexual behaviour Shortly after transfer find: * Men were 13% points more likely to engage in any vaginal sex (*p* < 0.01) * Men had 0.6 additional days of sex (*p* < 0.05) * Men reported using a condom more often during sex (6.9% points more likely; *p* < 0.05) * Women were less likely to report having any vaginal sex (9% reduction; *p* < 0.05) * Women had no change in reported condom use	
Minnis et al. [42]	Yo Puedo – conditional cash transfers for completion of educational and reproductive health wellness goal; life skills	USA	Youth 16–21 years of age and same-aged members of their social network	72 networks, 162 youth; randomised into intervention and control; 6-month follow-up	*Lower odds of having sex (OR 0.50; *p* = .04)

**Table 2.** Economic strengthening interventions to prevent HIV or IPV (1 January 2000–1 January 2015).

Reference	Intervention	Country	Population	Study design	HIV outcome	IPV outcome
Microfinance						
Ahmed [43]	Microfinance	Bangladesh	BRAC households, women	422 women in BRAC households, 1622 in non-BRAC households; cross-sectional; 60 BRAC-ICDDR villages		* Higher experience of IPV in BRAC households (*p* = 0.05) * Logistic regression suggests initially violence increases then subsides
Bajracharya [44]	Microfinance	Bangladesh	Women, Demographic Health Surveillance (DHS)data	4195; cross-sectional DHS; propensity score matching		* No difference in IPV experienced between microfinance and non-microfinance participants
Chin [45]	Microcredit	Bangladesh	Women DHS data	1843; cross-sectional DHS		* IPV reduces over time through participation
Dalal et al. [46]	Microfinance	Bangladesh	Women	4465 women; cross-sectional; nationally representative		* For women with secondary or higher education, and women at the two wealthiest levels of the wealth index, microfinance programme membership increased the exposure to IPV two and three times, respectively * The least educated and poorest groups showed no change in exposure to IPV associated with microfinance programmes
Murshid et al. [47]	Microfinance	Bangladesh	Women	4163 ever-married women, DHS		* Significant increase in IPV for women involved in microfinance who were better off (*p* = 0.008)
Koenig et al. [48]	Microcredit	Bangladesh	Women	10,368 currently married women; cross-sectional; 2 districts		Impact of involvement in microfinance on IPV context specific: * In conservative areas involvement increased vulnerability * In more liberal areas it had no impact
Naved and Persson [49]	Microfinance	Bangladesh	Women	2702 women; population-based survey; cross-sectional		* Increase in IPV in urban areas for belonging to credit group (OR 1.83; *p* < 0.01)
Schuler et al. [50]	Microfinance	Bangladesh	Women	1305; cross-sectional		* Membership of microfinance institutions showed significant reduction in IPV (*p* < 0.05)
Hadi [51]	Microfinance	Bangladesh	Women, under 50	500 women, cross-sectional		* Involvement in microcredit institution for more than 5 years reduces sexual violence (*p* < 0.1), similar to those not eligible
Bates et al. [52]	Microcredit	Bangladesh	Women	1212; cross-sectional		* Involvement in microcredit institution reduced IPV by 25% (*p* < 0.05)
Kim et al. [53]	Microfinance	South Africa	Women	1489 in intervention; 1647 in control; cross-sectional	* No impact on HIV risk behaviours	* No impact on IPV outcomes

**Table 3.** Economic strengthening and gender transformative interventions to prevent HIV or IPV (1 January 2000–1 January 2015).

Reference	Intervention	Country	Population		Study design	HIV outcome	IPV outcome
Microfinance/VSLA and gendertransformative							
Pronyk et al. [54]	Intervention for Microfinance for AIDS and Gender Equity (I.M.A.G.E) Project. Microfinance; Sisters for Life 10 sessions around gender and life skills	South Africa	Poorest women in communities, identified via participatory wealth ranking; av. age 41	8 clusters randomly allocated into intervention or control; main group-matched control of women 860		* Young women [14–34,37] less likely to have unprotected sex with non-spousal partner (a.R.R. 0.76)	* Reduction of IPV by 55% (a.R.R 0.45 [signif] amongst those directly involved)
Gupta et al. [55]	VSLA for women; 8 couples dialogue sessions	Ivory Coast	Women; av. age 37.7	934 women; 24 intervention clusters, 23 control clusters, randomly allocated; 12–18-month follow-up			* Reduced sexual and physical IPV (not signif) * Reduced economic abuse (O.R = 0.39, *p* < 0.0001) * Reduced acceptability of wife-beating (*p* = 0.006)
Spielberg et al. [56]	Existing self-help groups; 10 sessions around gender and life skills	India	Women participating in self-help groups; adolescents identified by women	Cluster randomised trial; 55 villages assigned to intervention (n = 32) or control (n = 23); 6- and 12-month follow-up		Women saw: * No impact on condom use	
IRC [57]	VSLA for women; 8 couples dialogue sessions	Burundi	Women members of VSLA	483 women; randomised into intervention and control; 15-month follow-up			* Women in the high or moderate risk category at baseline reported a 22% significant reduction in the incidence of violence in the last two weeks and a 46% reduction in physical harm (no significance reported)
Rosenberg et al. [58]	Microfinance; max 5 training sessions; health book	Haiti	Women; age: mean 36.1; range 18–49	192 participants; cross-sectional		* Improved condom use in last year: O.R 0.63 (95% C.I 0.26–1.54) * Improved condom use among those with an unfaithful partner: O.R 3.95 (95% C.I 0.93–16.85)	
Dunbar et al. [59]	SHAZ! 10 life skills modules; 5-day business training; microcredit loans	Zimbabwe	Adolescent female orphans out of school; 16–19; av. age 17.5	49 women; pre-test, post-test; 6-month follow-up; no randomisation		* Use condom with primary partner: post 38% vs pre 67% (*p* = 0.35) *Currently sexually active: post 22% vs pre 18% (*p* = 0.79) * Power in sexual relationship: post 50% vs pre 11% (*p* = 0.79) * Power in non-sexual relationship: post 38% vs pre 5% (*p* = 0.04)	
Bandiera et al. [60]	Life skills training; vocational training; microfinance	Uganda	Adolescent girls; av. age 16	4800 adolescent girls; 100 communities randomly assigned to intervention or control; 2-year follow-up		* Improved condom use (*p* < 0.05)	* 76% reduction in unwilling sex (*p* < 0.01)
Erulkar and Chong [61]	Modified microfinance; mentoring; life skills	Kenya	Girls out-of-school 16–22	326 matched pairs; interviews as exited programme – mixed follow-up time		* Condom use at last sex: at endline TRY = 52.1% vs control = 44.3% (N.S) * Able to refuse sex to spouse/partner: at endline TRY = 80.3% vs control = 71.6% (*p* < 0.05)	
Souverein et al. [62]	Comprehensive sex worker community mobilisation, including STI prevention and treatment, crisis support and microfinance	India	Female sex workers	17,092; using entry and exit from systems, 2005–2010; descriptive study		* Significant reduction in STIs (*p* < 0.001) *Increase in condom use (*p* < 0.001)	
Odek et al. [63]	Existing peer education intervention on HIV; microfinance	Kenya	Female sex workers; av. age: 41.09	227 women; 2-year follow-up; pre-test, post-test; no randomisation		* Reduction in number of sexual partners in past week (*p* < 0.001) * Reduction in number of casual partners (*p* = 0.098) * Reduction in number of regular partners (*p* < 0.001) * No impact on condom use with casual partners (*p* = 0.727) * Increase in condom use with regular partners (*p* = 0.031)	
Witte et al. [64]	34-session HIV sexual risk reduction intervention; microfinance; vocational training	Mongolia	Female sex workers; av. age 36	107 women randomised into intervention or control; clusters 3- and 6-month follow-up		* Reduction in number of paying sexual partners (*p* < 0.001) * 3.72 times more likely to report no unprotected vaginal sex acts at 6 months (*p* < 0.05)	
**Savings and gender transformative**							
Austrian and Muthengi [65]	Safe spaces; reproductive health training; financial education; savings accounts	Uganda	Girls 10–23; majority under 19		1159 of which 451 received full intervention; 300 savings only; 311 control; 12-month follow-up; delivery error led to natural randomisation		* No impact on indecent touching in full intervention, but in economic-only arm significant increase (*p* < 0.01)
Austrian and Muthengi [66]	Safe spaces; reproductive health training; financial education; savings accounts	Kenya; Uganda	Girls 10–19		1473 in Kenya; 1564 in Uganda; 18-month follow-up; comparison groups		* In Kenya no impact on indecently touched in past 6 months * Significant decrease in being indecently touched in past 6 months in Uganda
**Vocational strengthening and gender transformative interventions**							
Dunbar et al. [67]	SHAZ! reproductive health services; life skills-based HIV education; vocational training and microgrants; integrated social support	Zimbabwe	Adolescent female orphans (having lostat least one parent) aged 16 to 19		315 randomly assigned to intervention or control; 24-month follow-up	* No difference in sexual debut *Reduction in transactional sex (Intervention Odds Ratio [I.O.R] = 0.64; 95% C.I 0.50–0.83) * Increased likelihood of using condom (I.O.R = 1.79; 95% C.I 1.23–2.62)	* Reduction in violence (I.O.R = 0.10 vs Control Odds Ratio [C.O.R] = 0.63; *p* = 0.06)
Jewkes et al. [68]	Stepping Stones and Creating Futures; reproductive health and gender training; livelihood training	South Africa	Young men and women [18–29,31];av. age 22		232 participants; shortened interrupted time series design, 12-month follow-up	*Increase in men reporting last person they hadsex with was main partner (signif) *No impact on condom use at last sex *No impact on transactional sex	*Reduction in sexual violence experience by women (*p* = 0.033) *No impact on sexual or physical violence perpetrated by men
Rotheram-Borus et al. [69]	Street Smart: 10 sessions HIV prevention; vocational training of apprenticeships	Uganda	Age 13–23 years		100 participants; pre-test, post-test with random assignment to immediate intervention or delayed vocational training; 24-month follow-up	*No impact on mean number of partners *No impact on abstinence/100% condom use	
Sherman et al. [70]	JEWEL: HIV education; microfinance; vocational training	USA	Drug-using women who were involved in sex work; aged 18–45		54 women; baseline and 3-month follow-up	*Reduction in receiving drugs or money for sex (100% vs 71.0%; *p* < 0.0005) *Reduction in median number of sex trade partners per month (9 vs 3; *p* < 0.02)	
Sherman et al. [71]	Pi Bags: HIV education; vocational training	India	Female sex workers; age (median): 35		100 participants; randomised into intervention and control; 6-month follow-up	*Reduction in number of sex partners (*p* < 0.001) *Reduction in number of sex exchange partners (*p* < 0.001) *No impact on condom use at last sex exchange (*p* = 0.32)	
Lee et al. [72]	SiRCHESI Hotel Apprenticeship Program (HAP) participants; literacy; health education; life skills; vocational training; apprenticeships	Cambodia	Beer girls/sex workers; av. age: 24.93; range: 19–31		14 participants with baseline and 8-month and 24-month follow-ups	*Increase in condom use at last sex (*p* = 0.050)	
Raj et al. [73]	M.E.N (Making Employment Needs) Count HIV intervention; HIV, gender equity; employment and housing	USA	Men aged 18–54		50 participants; pre-test, post-test; 60–90 follow-up	*Unprotected sex decreased (*p* = 0.04) *No impact on multiple partners	*Too small at baseline to report trends
Hallman and Roca [74]	Siyakha Nentsha Programme: financial training, life skills and reproductive health training	South Africa	School-age boys and girls (14–16)		18-month follow-up; unclear sample or allocation	*Intervention boys significantly more likely to remain abstinent (no *p*-values reported)	

Thirteen separate analyses of six different cash transfer interventions were identified. They were separated into whether they subscribed to a broad social protection approach, or used a behavioural economics approach as their theoretical underpinnings [26].

(2) Social protection

Ten analyses of social protection interventions were identified on eight different interventions (Table 1); five focused only on child outcomes (all from Africa) and five focused only on adult women’s outcomes (all but one from Latin America).

(3) Child outcomes

Five analyses focused on two different interventions. In South Africa the CSG had three different analyses [30–32] and the Kenyan Cash Transfer for Orphans and Vulnerable Children (Kenya CT-OVC) had two different analyses from one study [33,34]. The Kenya CT-OVC [33,34]was an RCT, while one study of the South African CSG used a prospective cohort, with one analysis using propensity score matching to adjust for selection bias [30,31], while the other study of the South African CSG was a cross-sectional design [32].

All five social protection cash transfer evaluations focused on child outcomes (under 18) reported on HIV risk behaviours; none reported on under-18s’ experiences of IPV. All analyses of the South African CSG reported positive outcomes for children. Cluver et al. [30] reported a significant reduction in transactional sex and age-disparate sex for girls, but no impact for boys, amongst households receiving the CSG. Similarly cross-sectional analysis of the CSG by UNICEF reported a significant reduction in sexual activity, delayed sexual debut and reduced pregnancies for both girls and boys whose families received the CSG [32]. Cluver and colleagues [31] undertook a separate analysis of cash combined with care (defined as either a supportive teacher or positive parenting at home), and found significant reductions in HIV risk behaviour for girls and boys, where families received the CSG. In Kenya, Handa et al. [33] reported a significant delay of sexual debut for children receiving cash; however, Rosenberg et al. [34] reported no impact of the transfer on transactional sex or relative partner age. They do suggest that there were marginal reductions in transactional sex if the girl was younger or in school [34].

(4) Adult outcomes

Five separate analyses of four different broad-based social protection interventions were identified; all reported adult women’s outcomes. All focused exclusively on women’s self-reported experiences of IPV, without consideration of HIV outcomes, with four from Latin America and one from Kenya. Two analyses focused on Mexico’s Oportunidades, which is a conditional cash transfer linked to accessing health and education services [37,38]; one focused on Bono de Desarrollo Humano (B.D.H), a state-run unconditional cash transfer in Ecuador [35]; and one focused on a humanitarian relief intervention in Ecuador, which operated for six months [75]. The final study was of an unconditional cash transfer called ‘GiveDirectly’ run by an NGO in rural Kenya, which provided either a lump sum or regular transfers of a smaller amount over nine months [39]. Study quality was generally high with randomised designs in Kenya and Ecuador [35,36,39]. Two studies, both from Mexico, were cross-sectional in design [37,38].

All analyses focused on women’s self-reported experience of IPV. Two studies reported reductions of physical and sexual IPV. In Kenya the GiveDirectly intervention showed a 30–50% reduction in various forms of physical violence and a 50% reduction in rape (*p* < 0.1) and 60% reduction in other forms of sexual violence (*p* < 0.05) [39]. Similarly, in Ecuador, the short-term transfer intervention showed reductions in controlling behaviours and women’s experiences of IPV [75].

In contrast, other studies reported mixed results. In Mexico, one analysis of Oportunidades showed mixed outcomes for women receiving a cash transfer: while it reduced physical IPV by 40% (*p* < 0.01), it increased women’s experiences of emotional violence by 3 to 5 percentage points, but this change was not statistically significant [35]. In the BDH evaluation no impact was seen on reducing violence, while an increase in male controlling behaviours was reported (*p* < 0.05) [35]. Another study showed no long-term impact on IPV; however, analyses suggested this was because women could leave abusive partners because of receipt of the transfer [38].

(5) Cash transfers as incentive for behaviour change

Three cash transfer interventions were based in behavioural economics, aiming to shift health behaviours and thus HIV- or STI-status. Two studies were conducted in sub-Saharan Africa (SSA) [40,41] and one in the USA [42]. In general study design was rigorous, using RCTs in two studies [40,41] and a quasi-experimental design in the other [42]. In Tanzania remaining STI-free was incentivised with cash and assessed through testing every 4 months over a 12-month period [40]. In Malawi a study focused on incentivising maintaining HIV status over 12 months for women and men (if HIV-negative at start, maintaining this; if HIV-positive, they automatically received the cash transfer at the end) [41]. Finally, in the USA, a study provided cash transfers on progression through a series of health checks and training programmes [42]. All three studies focused on women and men, 16 years old and older.

There was mixed evidence on the impact of incentivising HIV-status or STI-status. In Tanzania, adjusted analysis showed significant impacts of high-value incentives on remaining STI-free [40]. In Malawi, however, there was no impact on maintaining HIV-status [41]. But, when women and men were given the incentive, HIV risk behaviours reduced for women and increased for men [41]. In the USA, the combination of incentives linked to accessing services and progression in training programmes reduced the likelihood that participants had sex [42].

1. Economic strengthening interventions

Eleven studies sought to assess the impact of economic strengthening interventions on IPV and HIV-prevention (Table 2). Ten were from Bangladesh and one from South Africa. All studies focused on the impact of microfinance and used cross-sectional study designs. Given study designs – with many looking at population-level data – it is difficult to untangle whether similar interventions were assessed. In Bangladesh Bajracharya [44] used propensity score matching on cross-sectional data to overcome bias. All studies included measures of IPV experienced by women; one study also included measures of HIV risk behaviours [53].

Overall there was no clear evidence on the impact of microfinance on women’s experiences of IPV. Two studies – one from Bangladesh and one from South Africa – suggested women’s participation in microfinance had no impact on IPV [44,53], while two studies from Bangladesh suggested involvement in microfinance reduced IPV [50,52], with three others suggesting a potential initial increase in women’s experience of IPV, and a reduction in risk over a longer time period [43,45,51]. In contrast, the other four studies suggested involvement in microfinance increased IPV, particularly under specific circumstances, including living in a conservative area [48], being wealthier [46,47,49] and residing in urban areas [49], suggesting a significant role was played by contextual factors. The only study which looked at HIV risk behaviours cross-sectionally in South Africa found no impact on these [53].

1. Economic strengthening and gender transformative

We identified 21 interventions, each with their own separate analysis, which combined economic strengthening interventions with gender transformative components, primarily group-based discussions, and couples interventions (Table 3). Three main types of economic interventions were identified: microfinance/Village Savings and Loans Association (VSLA), formal savings, and vocational training. We report under each of these types separately.

1. Microfinance/VSLA and gender transformative

Eleven interventions used microfinance or VSLA. Seven studies were undertaken in SSA, three in Asia and one in Latin America. Study design varied: six were RCTs [54–57,60,64], four were quasi-experimental [59,61–63,76] and one was cross-sectional [58]. The additional gender transformative components varied widely. Two used couples discussion groups [55,57], while another study was embedded in a wider community mobilisation intervention for sex workers [62]. The others all worked with women in group settings, such as VSLAs. Nine reported HIV outcomes and four IPV outcomes (only two reported both [54,60]).

In general, despite variations in reported outcomes, there was a trend towards positive outcomes. For HIV-related outcomes, six reported significant improvements in condoms use [23,58,60,62–64], while two reported non-significant improvements [59,77] and one reported no change [56]. Other changes in reported HIV risks included a reduction in STIs [62] and a reduction in the number of sexual partners [63,64], all in sex worker populations.

Four studies reported IPV-related outcomes [54,55,57,60]. Three reported significant reductions on IPV [23,57,60] and one a non-significant but positive outcome [55]. One study also reported a reduction in economic abuse from their partner (*p* < 0.0001) in female participants [55].

1. Savings and gender transformative

Only one discrete intervention was identified that reported a behavioural outcome in two studies [65,66]. This focused on young women and included a pilot from two countries (Kenya and Uganda) using a pre-test, post-test design [66] and a subsequent larger study from Uganda, with three arms: full treatment, economic-only treatment and comparison group [65]. The full intervention provided safe social spaces, reproductive health training, financial education and opened a savings account for children [65,66]. Only IPV outcomes data were reported.

The outcomes of this saving and gender transformative intervention were mixed. In the pilot there were limited effects seen in Kenya, but in Uganda there was a significant decrease in being indecently touched by anyone (whether a partner or non-partner) in the past six months [66]. In the subsequent controlled study, the full intervention showed no impact on sexual violence; however, in the economic-only arm, there was a significant increase in indecent touching by anyone in the past six months (*p* < 0.01) [65].

1. Vocational training and gender transformative

Eight interventions provided vocational training combined with gender transformative training [59,68–74]. Four were in SSA, two in Asia and two in the USA. One study was an RCT, although randomisation occurred at the individual, rather than group, level [67]. All others were quasi-experimental, although some did include elements of randomisation. All eight reported on a variety of HIV-related outcomes, however only three reported on IPV as well [67,68,73].

Vocational training varied. For instance, in the JEWEL intervention, Sherman et al. [70] provided jewellery-making training; SHAZ! provided options including hairdressing, garment making and receptionist training, alongside broader business skills [67]; while in the MEN Count intervention [73] employment counselling was provided. In contrast, in Stepping Stones and Creating Futures [68] there was no specific skills training, but rather the intervention focused on promoting critical thinking and reflection around livelihoods and job opportunities. There was a greater focus on younger people in these programmes, with four interventions (out of eight) targeting youth participants [10–24] and typically both women and men were included [67–69,74].

There were significant variations in HIV-related outcomes. Seven studies reported positive outcomes in terms of HIV-related ones, although the specific impact varied. In three studies condom use improved significantly [67,72,73]. In five studies, there were significant changes in other types of sexual behaviour or partner types, suggesting a reduction in HIV risk behaviour [59,68,70,71,74]. Only one study reported entirely no impact on behavioural measures related to HIV risk [69].

In the three studies reporting IPV, all reported reductions in IPV. Dunbar et al. [67] reported a non-significant reduction in IPV (IOR = 0.10 vs COR = 0.63; *p* = 0.06) for SHAZ!, while Jewkes et al. [68] reported a significant reduction in any sexual IPV experienced by women (37%; *p* = 0.033), but did not see a reduction in men’s perpetration of IPV for Stepping Stones and Creating Futures. The MEN Count intervention had too small a sample at baseline to report IPV trends, but suggested there was a non-significant reduction [73].

## Discussion

This is the first study to undertake a comprehensive review of any types of economic intervention which report either an HIV-related and/or IPV behavioural outcome. We identified 45 separate analyses of interventions that sought to use economic interventions (either on their own or in combination with other interventions) to prevent HIV risk behaviours and/or IPV. Interventions varied substantially in how they conceptualised the role of economics in preventing HIV or IPV, in study design, and in measurement. As such there was too much heterogeneity to undertake a meta-analysis of outcomes. However, the broad scope of the review enables some assessments of the field of interventions to be made.

The provision of cash through broad-based social protection programmes appears to have broadly positive outcomes for children targeted through these interventions. In the five separate analyses that looked at the impact of providing cash transfers to families with children, the results were positive in four of the studies in reducing HIV risk behaviours of children [30–33] and showed no impact in the fifth [34]. This is highly suggestive that, following a range of other reviews, the provision of broad-based social protection mechanisms as an approach to improving child health has multiple and overlapping positive outcomes [26,78]. However, no study looked at the potential impact of social protection on reducing children’s vulnerability to IPV as they moved into adolescence.

For adults, however, interventions that solely strengthened economic well-being, through either cash transfers, or involvement of adults in economic strengthening activities such as microfinance or VSLA approaches, showed mixed outcomes, with studies reporting increases, decreases and no impact on HIV risk behaviours and IPV. The ‘mixed’ findings of these interventions can be explained in a number of ways. First, some studies have emphasised that as women gain economic autonomy and power in relationships more generally, they may face a ‘male-backlash’ [79], as men start to feel their ‘authority’ is challenged. In India, a prospective cohort study of married women found that those who secured work in the study period were more likely to experience IPV than those who remained unemployed [80]. Second, studies reviewed highlighted that the impact of economic strengthening interventions on IPV may be contextually specific, with contextual factors including urban or rural residence [49] and whether the community was more liberal or conservative [48] shaping outcomes. Similarly, Heise and Kotsadam [81] in their cross-country analysis highlight that women’s work is protective for IPV in contexts where many women work, but increases IPV-vulnerability if few women work.

In contrast, the interventions which combined economic and gender transformative interventions showed positive or flat results, and no negative findings. This highlights the fact that women’s experiences of HIV- and IPV-vulnerability are often shaped at the intersection of gender inequalities and economic marginalisation [79] and that successfully working to reduce these risks needs to combine economic and gender transformative interventions [82].

The review highlighted a lack of evidence around effective interventions for a number of specific populations: in particular female adolescents, female sex workers and men. In general, interventions tended to focus on adult women, with only 14 out of the 45 analyses including women under the age of 18 (of which 5 were child outcomes for broad-based social transfer programmes). The lack of inclusion of women and girls under the age of 18 has been noted in wider reviews of women’s economic empowerment interventions [83]. The lack of inclusion of younger women emerges from a confluence of factors including lack of recognition of their vulnerability to HIV and IPV, ethical challenges of working with younger people, and resistance to seeing adolescent girls as autonomous actors.

Only three interventions specifically focused on female sex workers, despite global evidence on the overwhelming burden of HIV and violence in this population [84,85]. Overall, sex worker interventions showed positive results, and were often embedded in wider sex worker mobilisation interventions, rather than being ‘stand-alone’ interventions. The potential of wider structural and social change through community mobilisation, as with sex worker mobilisation, is an important avenue for further consideration. In addition, there remains an ongoing debate about definitions of sex work and transactional sex, especially for women under 18, where women who do not identify as sex workers may be excluded from sex worker mobilisation and interventions.

The lack of research around interventions for these two populations (women under 18 and female sex workers) is critical not only because of the high burden of HIV and IPV they face, but also because it is likely they face specific programming challenges. Indeed, the first generation of interventions with adolescent girls and young women showed many of the challenges, with flat or underwhelming outcomes [59,61]. Yet as lessons have been learnt and interventions have been modified, programmes with adolescent girls are increasingly showing positive outcomes [67,68]. Similarly, a review by the Global Network of Sex Work Projects of economic interventions for female sex workers in Africa highlighted the importance of involving sex workers in the design and implementation of projects [86], suggesting that there needs to be significant investment in developing and evaluating economic interventions for sex workers and that there is no simple transferability of interventions from one setting to another.

There was also a lack of focus on the potential and pitfalls of including men in economic and gender transformative interventions. Only 11 interventions out of 41 directly targeted men as recipients of the intervention and/or evaluated the impact on them. That there is little focus on including men in evaluations is a challenge for three reasons. First, men are already included in large-scale public works programmes across the world that seek to build their economic livelihoods and little is known about the impact of involving these men on HIV risk behaviours and IPV perpetration. Second, studies working with men for gender equality continually raise the challenge of poverty and the barriers this causes for transformation of masculinities [87] and as such, there may be significant benefit in including men in combination gender transformative and economic strengthening interventions. Third, given the potential for a ‘male-backlash’ around women’s involvement in economic strengthening interventions, understanding men’s responses remains critical. Further research and theorising need to be done to understand how working with men on strengthening livelihoods can be done in a way that promotes gender equality and does not lead to the exclusion of women [88].

The review also highlights missed opportunities in understanding the impacts of interventions at the intersections of HIV and IPV; only 6 out of 45 studies measured both HIV risk behaviours and IPV outcomes. Given the clear evidence of how IPV and HIV have common risk factors, the failure to include both sets of measures is a major evidence gap. Moreover, studies also point to the need to include a wider range of measures. For instance some studies pointed to the potential of economic-based interventions to either increase or decrease male controlling behaviours [35,36,68], an important factor for HIV acquisition and IPV [89], but this measure was not common across many studies. In addition, economic violence was only measured in a limited number of studies [55], yet given the underlying aim of some economic interventions is to increase women’s economic autonomy in relation to male partners, this is similarly a missed opportunity.

This review has a number of limitations. There is a widely known publication bias whereby positive results are much more likely to be published [90], which while somewhat mitigated by the inclusion of grey material, is unlikely to have been totally overcome. Additionally, as any quantitative evaluation was included, irrespective of the quality of the study design, comparing different studies to each other may misrepresent interventions’ true effectiveness. Finally, as the review was not a full systematic review following Preferred Reporting Items for Systematic Reviews and Meta-Analyses (PRISMA) guidelines, there are likely to be differences between this review and a PRISMA-guided one.

This comprehensive review of economic interventions highlights the large evidence base that exists around reducing HIV risk and IPV globally through tackling poverty through a variety of interventions. It is highly suggestive that broad-based cash transfer interventions have widespread positive benefits for women who receive them, as well as their children targeted. In addition, it emphasises the positive outcomes of combining economic strengthening and gender transformative interventions. However, it also highlights the need for further research on this topic, including research on specific populations, female adolescents, female sex workers and men, if a full understanding of the benefits of these interventions is to be achieved.

## Paper context

Intimate partner violence and HIV are co-occuring global epidemics. Reviews have not explored the impact of economic interventions on both of these. We undertook a comprehensive review of these interventions. The review highlights that economic interventions combined with gender transformative components have stronger outcomes than economic interventions alone; there is a lack of meaningful inclusion of sex workers, young women and men in evaluations; and there are missed opportunities for measuring HIV risk and intimate partner violence.

## Appendix A

**Table:** The search string used for the review was as follows.

Intervention	cash transfer*; cash incentive*; cash reward*; monetary reward*; economic asset*; contingency management; micro-credit; micro credit; microcredit; job training; income generation; income generating; job skills; employment; economic empowerment; cooperatives; microfinance; micro finance; microfinance; micro-enterprise; micro enterprise; microenterprise; small business; small loans; microloans; vocational training; business training; livelihood*
HIV	HIV; acquired immunodeficiency syndrome; acquired AND immunodeficiency AND syndrome;
IPV	domestic violence; family violence; sex offences; sexual violence; battered women; spouse abuse; IPV; intimate partner violence; VAW; violence against women; economic violence; emotional violence; physical violence; anti-violence; anti violence; antiviolence; gender-based violence; gender based violence; GBV; domestic violence; abused women
